# Syphilis infection is associated with an increase in plasma viral load in HIV infected patients: results from the FHDH cohort — ANRS CO4

**DOI:** 10.1186/1758-2652-13-S4-P223

**Published:** 2010-11-08

**Authors:** W Jarzebowski, C Piketty, P de Truchis, E Caumes, D Farhi, AS Lascaux, MA Bouldouyre, O Derradji, J Pacanowski, N Dupin, D Costagliola, S Grabar

**Affiliations:** 1 Hopital Cochin, Biostatistic and Epidemiology, Paris, France; 2 Hopital Europeen Georges Pompidou, Clinical immunology, Paris, France; 3 Hôpital Raymond Poincaré, Paris, France; 4 Hopital Pitie Salpetrière, Maladies infectieuses, Paris, France; 5 Hopital Tarnier, Paris, France; 6 Hôpital Henri Mondor, Créteil, France; 7 Hôpital St Louis, Paris, France; 8 Hôpital Paul Brousse, Villejuif, France; 9 Hôpital St Antoine, Paris, France; 10 Hôpital Tarnier, Paris, France; 11 Inserm U943, 56 Bd Vincent Auriol, BP 335, Paris, France

## Background

The effect of syphilis on HIV infection remains controversial. Most studies involved small sample sized population and did not account for cART effects on HIV markers.

## Purpose of the study

To assess the impact of syphilis infection on plasma HIV RNA (pVL) and CD4 cell counts.

## Methods

In HIV-infected men followed in Paris between 1998 and 2006 in the FHDH cohort, we studied 2 matched groups of patients: the syphilis+ and syphilis-. The syphilis+ group consisted of men who were diagnosed with an incident primary or secondary syphilis during their HIV follow-up. Each syphilis+ patient was matched up to 5 men who did not contract syphilis (syphilis-) according to his age, sexuality, centre and date of syphilis diagnosis (index date), and to his immunologic and virologic status in the period prior to syphilis infection. We studied whether syphilis infection was associated with an increase in pVL in the 6 months following infection (rise of pVL ≥0.5 log or pVL ≥500 copies/mL in patients with prior undetectable pVL) by conditional logistic regression. Changes in CD4 cell counts were studied by linear mixed model.

## Results

282 syphilis+ (64 primary and 218 secondary) and 1233 syphilis- patients were included. 89% of the patients were MSM aged 38 years in median. 86% were on cART at the index date and 17% had a previous AIDS diagnosis. Median CD4 cell counts before syphilis was 480/mm3, 58% of the patients had pVL<500 copies/mL. In the 6 months after infection, 40(14.2%) syphilis+ and 84 (6.8%) syphilis- patients exhibited a rise in pVL. Compared to syphilis- patients, syphilis+ patients had a higher risk of pVL increase (adjusted OR; 2.30 IC95%,1.38-3.15). No statistical difference (p=0.20) was observed between syphilis patients with and without cART at the time of syphilis, (aOR; 1.89 IC95%,1.16-3.08) and (aOR; 3.42 IC95%,1.59-7.37). Compared to the syphilis- group, the level of CD4 cell count in the syphilis+ group dropped of -28 CD4/mm^3^ (p=0.001) during the episode of syphilis but did not differ significantly after the episode (-3 cells/mm^3^, p=0.78). No change in the CD4 slopes was evidenced after the episode in both groups.

## Conclusions

In this large prospective cohort study with adjustments for age and treatment, syphilis infection was associated with a transient drop in CD4 cell count, which was significantly regain at the end of the episode but exposed patients to a higher risk of increase of viral load.

